# Multi-frequency analysis of brain connectivity networks in migraineurs: a magnetoencephalography study

**DOI:** 10.1186/s10194-016-0636-7

**Published:** 2016-04-18

**Authors:** Di Wu, Yuchen Zhou, Jing Xiang, Lu Tang, Hongxing Liu, Shuyang Huang, Ting Wu, Qiqi Chen, Xiaoshan Wang

**Affiliations:** Department of Neurology, Nanjing Brain Hospital, Nanjing Medical University, 264 Guangzhou Road, Nanjing, Jiangsu 210029 China; MEG Center, Division of Neurology, Cincinnati Children’s Hospital Medical Center, 3333 Burnet Avenue, Cincinnati, OH 45220 USA; MEG Center, Nanjing Brain Hospital, Nanjing, Jiangsu 210029 China

**Keywords:** Migraine, Magnetoencephalography (MEG), Multi-frequency, Functional connectivity, Neural network, Graph theory

## Abstract

**Background:**

Although alterations in resting-state neural network have been previously reported in migraine using functional MRI, whether this atypical neural network is frequency dependent remains unknown. The aim of this study was to investigate the alterations of the functional connectivity of neural network and their frequency specificity in migraineurs as compared with healthy controls by using magnetoencephalography (MEG) and concepts from graph theory.

**Methods:**

Twenty-three episodic migraine patients with and without aura, during the interictal period, and 23 age- and gender-matched healthy controls at resting state with eye-closed were studied with MEG. Functional connectivity of neural network from low (0.1–1 Hz) to high (80–250 Hz) frequency ranges was analyzed with topographic patterns and quantified with graph theory.

**Results:**

The topographic patterns of neural network showed that the migraineurs had significantly increased functional connectivity in the slow wave (0.1–1 Hz) band in the frontal area as compared with controls. Compared with the migraineurs without aura (MwoA), the migraineurs with aura (MwA) had significantly increased functional connectivity in the theta (4–8 Hz) band in the occipital area. Graph theory analysis revealed that the migraineurs had significantly increased connection strength in the slow wave (0.1–1 Hz) band, increased path length in the theta (4–8 Hz) and ripple (80–250 Hz) bands, and increased clustering coefficient in the slow wave (0.1–1 Hz) and theta (4–8 Hz) bands. The clinical characteristics had no significant correlation with interictal MEG parameters.

**Conclusions:**

Results indicate that functional connectivity of neural network in migraine is significantly impaired in both low- and high-frequency ranges. The alteration of neural network may imply that migraine is associated with functional brain reorganization.

## Background

Migraine is characterized by episodic headache attacks and is frequently accompanied by nausea, vomiting, yawning, photophobia, and phonophobia [1]. Our conceptualization of migraine has evolved from primarily a vascular disorder to a neurovascular disorder and currently to a brain disorder, primarily a disorder of neurons rather than a disorder of blood vessels [2, 3]. Numerous neuroimaging studies have used functional connectivity analysis to detect alterations in brain function in migraineurs and have revealed that neuroimaging alterations correlate with longer migraine duration and increased migraine frequency [4–8].

Functional connectivity has been considered an approach to describe how brain regions communicate information with each other [9]. During the resting state, correlated spontaneous fluctuations occur within spatially distinct and functionally related groups of cortical and subcortical regions, consisting of the human brain’s functional connectivity networks [10]. Functional connectivity measures the statistical interdependencies between two time series (brain signals), and different functional connectivity patterns reflect different models that brain regions work together [11]. The introduction of graph theory to neuroscience allows us to quantify different networks and further characterize the features of brain functional topology [12]. According to concepts from graph theory, the brain can be considered a mathematical network consisting of a set of nodes and links. Nodes in large-scale brain networks represent brain regions, and connections signify the communication among distinct brain regions (i.e., functional connectivity [13, 14]). Subsequently, graph theory has been extensively used to gain insight into how brain regions coordinate to support higher cognitive functions [15].

Electroencephalography (EEG) has been used in the study of functional connectivity or neural network in migraine [16–18]. By analysis of EEG signals in 0.5–30 Hz, de Tommaso and colleagues have found that migraineurs have increased connectivity between the bilateral temporal-parietal and the frontal regions around the midline as compared with healthy controls after laser stimulation [16]. It has been shown that neural network can reproduce the abnormality of the neurophysiological dysfunction in migraine [17]. An EEG study has demonstrated that MwA and MwoA are associated with different patterns of functional and effective connectivity in ongoing EEG under repetitive photic stimulation [18]. Functional magnetic resonance imaging (fMRI) study of migraine has also extended the focus from a single region or structure to a network of regions and structures and the interactions among them [19–21]. Tedeschi and colleagues have found that migraineurs exhibit an increased resting-state visual network connectivity, which may be related to the initiation and propagation of the migraine aura [19]. Mickleborough and colleagues have revealed that migraineurs lack suppression of unattended events and have heightened orienting to sudden onset stimuli in peripheral locations [20]. By using effective connectivity analysis of resting-state fMRI, Ting and colleagues have indicated that the dysfunctional ascending and descending pain network at the thalamic-level may play an important role in the study of migraine [21]. Though previous EEG and fMRI studies revealed the alteration of neural network in migraine, it remains unclear if the neural network impairment in migraine is frequency-dependent.

Magnetoencephalography (MEG), a relatively new clinical neuroimaging modality, is well suited for noninvasively detecting and localizing neuromagnetic signals [22]. MEG is considered to be superior to scalp EEG because the skull, skin, and other tissues can distort electric signals, whereas magnetic signals can pass through these tissues without significant distortion [23, 24]. Analysis of functional connectivity with MEG can investigate the functional organization of the brain based on temporal correlations in neuromagnetic signal fluctuations in different brain regions. Previous studies have used MEG with graph theory to reveal the features of the intrinsic functional network of the brain [25]. The changed features during the resting state may serve as a marker to reflect the progress of multiple diseases, such as Alzheimer’s disease [12], Parkinson’s disease [26], and Autism spectrum disorder [27]. Combining multi-frequency MEG analysis with graph theory, the neural network topology and its frequency specificity can be systematically demonstrated in both low- and high-frequency ranges.

The aim of this study was to investigate whether the functional connectivity neural networks are abnormal in migraineurs relative to healthy controls in low- and high-frequency ranges and whether the neural networks are different between the MwA and MwoA.

## Methods

This study was approved by the Institutional Review Board at Nanjing Brain Hospital. All subjects signed a consent form after the experimental procedures were fully explained.

### Subjects

The inclusion criteria for patients with migraine were based on the International Classification of Headache Disorders Second Edition (ICHD-II) [28, 29]. Exclusion criteria included the following: (1) existence of other neurological disease; and (2) use of prescription medications within a week prior to the study. Healthy controls were recruited to match subjects in the migraine group in terms of age and gender. Inclusion criteria included the following: (1) no history of any neurologic disorder; and (2) no first-degree relative with a history of any type of migraine. Exclusion criteria for all participants included the following: (1) presence of an implant (e.g., braces or pacemaker), which could produce visible magnetic noise in MEG data; (2) demonstration or expression of noticeable anxiety and/or inability to communicate readily with personnel operating the MEG equipment; (3) inability to keep still; and (4) pregnant patients or those with claustrophobic tendencies (for MRI scans).

Twenty-three patients with episodic migraine with or without aura (15 females; mean age: 30.04 years, standard deviation: 6.62 years, range: 20–40 years) were recruited from Nanjing Brain Hospital (Table 1). Ten (six females; mean age: 31.50 years, standard deviation: 7.52 years, range: 20–40 years) of 23 patients with migraine had aura. Thirteen (nine females; mean age: 29.31 years, standard deviation: 5.02 years, range: 20–36 years) of 23 patients with migraine had no aura. All patients with migraine were right handed. Twenty-three age- and gender-matched, right-handed healthy controls (15 females; mean age: 29.83 years, standard deviation: 6.86 years, range: 20–40 years) were recruited. Patients had no migraine headache attack during MEG recording. All subjects were headache free at least 72 h (h) prior to the testing and 24 h after the scan.

**Table 1:** Demographic and Clinical Characteristics of Migraineurs

Patients	Sex	Age (years)	Aura	History (years)	Attack Frequency	Duration (hours)	Pain Type	Pain Loacation	Pain Intensity	Onset to scan(days)
1	Female	27	No	10	2–3/month	24	Throbbing	Unilateral	5	5
2	Male	23	No	13	4/month	10	Constant	Unilateral	7	6
3	Female	36	No	12	1/month	20	Throbbing	Bilateral	7	4
4	Female	39	Yes	30	1/month	6	Pressure	Unilateral	6	15
5	Male	20	Yes	5	1–2/month	6	Pressure	Bilateral	7	7
6	Female	40	Yes	30	1–2/month	36	Throbbing	Unilateral	8	7
7	Female	34	No	12	2/month	4	Sharp	Unilateral	3	4
8	Female	40	Yes	16.5	1–2/month	48	Throbbing	Bilateral	7	5
9	Female	40	Yes	18	1–2/month	12	Pressure	Bilateral	7	10
10	Male	26	Yes	8	2/month	4	Throbbing	Unilateral	3	20
11	Female	26	No	3.5	3/month	72	Throbbing	Unilateral	8	5
12	Female	30	Yes	4.5	1–2/month	12	Pressure	Bilateral	6	4
13	Female	27	Yes	3.5	4/month	36	Constant	Unilateral	6	6
14	Female	32	No	2	1/month	48	Constant	Unilateral	5	7
15	Female	34	No	7	2/month	1	Pressure	Unilateral	6	7
16	Male	30	No	5.5	1/month	5	Pressure	Bilateral	3	4
17	Female	31	No	10	1–2/month	1.5	Stabbing	Unilateral	6	3
18	Male	24	No	7.5	1/month	12	Pressure	Unilateral	7	10
19	Male	20	No	4.5	3/month	12	Throbbing	Bilateral	5	6
20	Male	27	Yes	7	1/month	8	Pressure	Unilateral	7	5
21	Female	29	No	8	2–3/month	1	Squeezing	Unilateral	6	3
22	Female	35	No	10	1–2/month	2	Throbbing	Bilateral	7	4
23	Male	26	Yes	12	1/month	12	Throbbing	Unilateral	5	3

### MEG recording

MEG signals were recorded in a magnetically shielded room by using a whole-head CTF 275-Channel MEG system (VSM MedTech Systems, Inc., Coquitlam, BC, Canada) in the MEG Center at Nanjing Brain Hospital. Before data acquisition, every subject was asked to remove all metals from their body. Electromagnetic coils were attached to the nasion and to the left and right preauricular points of the subject. These three coils were subsequently activated at different frequencies to measure the head positions of the subject relative to the MEG sensors. MEG data were recorded at a sampling rate of 6000 Hz for 120 s. Subjects were placed in the supine position and instructed to keep their eyes closed during the recording, not to think of anything in particular, and not to fall asleep. After scanning, the subjects were asked if they remained awake during the whole procedure. The head positions were measured at the beginning and end of the scanning. If head movement during recording was beyond 5 mm, the dataset was indicated as “bad” and an additional dataset was recorded.

### MRI scanning

Anatomical image data were recorded for all subjects by using a 1.5 T MRI scanner (Singa, GE, USA). Three fiducial points were placed at the same locations to facilitate co-registration of MEG and MRI data; these points were regarded as the positions of the three coils used in the MEG recordings before MRI scan. Subsequently, all anatomical landmarks digitized in the MEG study were identified during MRI scanning.

### Data preprocessing

**Fig. 1:**
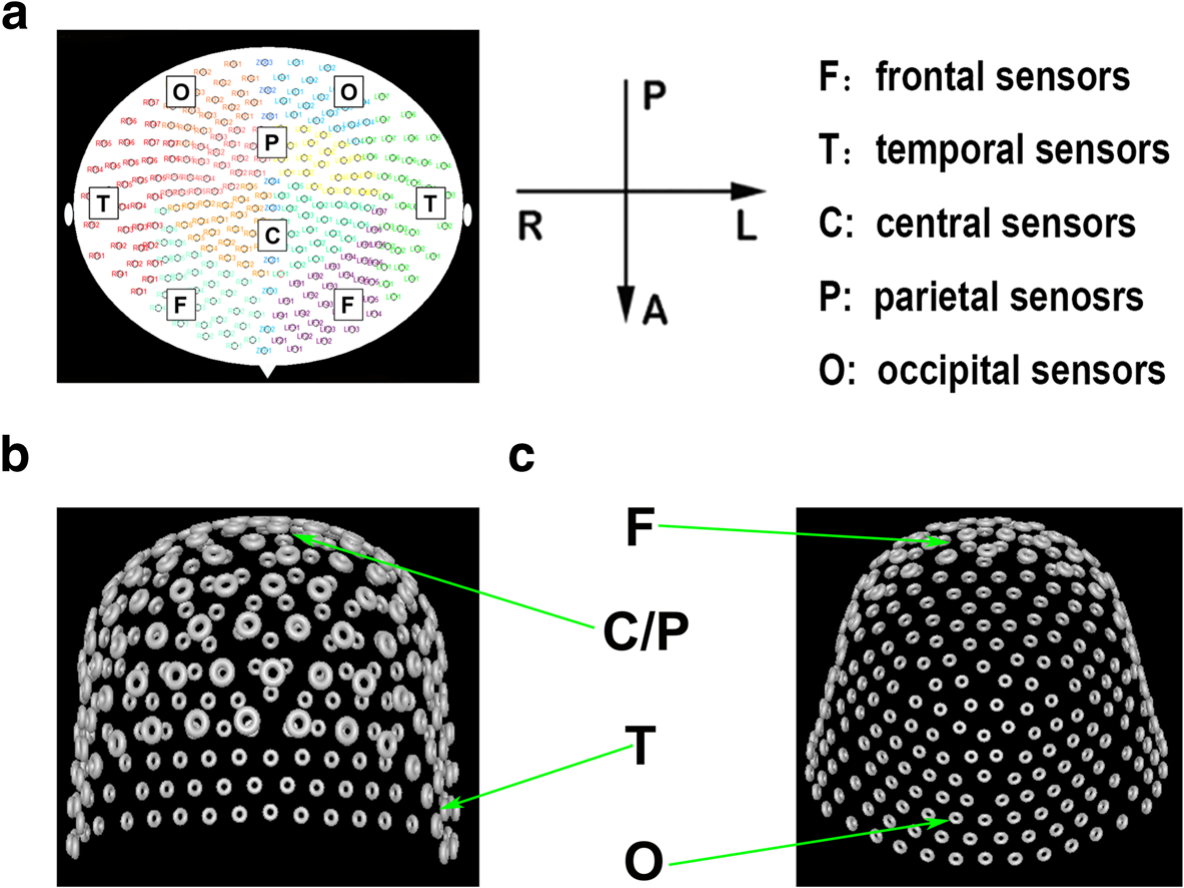
Distribution map of MEG sensors. **a** 2D map of the distributionof all MEG sensors. **b** 3D front view of the distribution of all MEG sensors. **c** 3D bottom view of the distribution of all MEG sensors

Similar to previous reports [30, 31], MEG waveforms were visually inspected for identifying magnetic noise and other artifacts. Any MEG data with noticeable magnetic noise or artifact (>6 pT) were excluded from the analyses. The MEG data were preprocessed by removing the direct current offset. The neural network at the sensor levels was analyzed by computing the coherence of MEG signals in multiple regions. A custom-designed program, MEG Processor, was used to compute and visualize functional networks at the sensor level [32, 33]. Neural activity (120 s) was filtered into seven frequency bands: slow wave (0.1–1 Hz), delta (1–4 Hz), theta (4–8 Hz), alpha (8–12 Hz), beta (12–30 Hz), gamma (30–80 Hz), and ripple (80–250 Hz). The distribution of the coherence for each possible pair of the 275 sensors was displayed in topographic patterns (network contourmaps, see Fig. 1). Red color indicated excitatory connection on contourmaps. An excitatory connection was a positive connection where the amplitude of signals in two connected sensors was positively correlated. Blue color indicated inhibitory connections on contourmaps. An inhibitory connection was a negative connection where the amplitude of signals in a sensor pair was negatively correlated. To compare migraineurs and controls, the same threshold value was used to exhibit contourmaps for all subjects.

Graph theory analysis [25] was performed to quantify the functional connectivity of networks at the sensor level. Neural networks were identified based on correlations among MEG signals from different brain regions. A total of 275 MEG sensors were considered a collection of nodes, whereas edges corresponded to functional connections between all pairs of these nodes. In this study, we focused on the network parameters of connection strength, path length, and clustering coefficient [13]. Average connection strength refers to the average functional connectivity between all possible connections of the entire MEG sensor array. The average path length refers to the average path length over each possible pair of nodes. This parameter is a measure of global integration of the network. The clustering coefficient of a node represents the ratio of all existing connections between the “neighbors” of a node (nodes that are one-step away) and the maximum possible number of edges between the neighbors. The average clustering coefficient of a network indicates the average clustering value over each node and represents a measure of the tendency of network elements to form local clusters.

### Statistical analysis

Fisher’s exact tests were used to analyze the odds ratio of functional connections in brain areas among the migraineurs and the controls. Analysis of variance analysis was applied to the power value difference between the migraineurs and the controls. Post-hoc comparison was conducted with two-tailed Student *t*-test for assessing the network parameters (strength, path length and clustering coefficient) between the migraine and control groups. The correlations between migraine clinical characteristics (age, headache history, attack frequency, duration, pain intensity and time from the last attack) and MEG parameters (topographic patterns of neural network and network parameters) were analyzed using Spearman correlation coefficients. A significance level of 0.05 (two-tailed) was accepted for each comparison. If multiple testing was considered, the significance level for each of these tests was reduced from 0.05 to 0.016 (three parameters, Bonferroni correction). Statistical analyses were performed using SPSS version 19.0 software package (IBM Inc.).

## Results

### Clinical characteristics

Of 23 patients with migraine, 15 (65 %) were females (eight were males, 35 %). Out of the 23 patients, 10 (43 %) had visual aura, 20 (87 %) presented moderate to severe headache, and 15 (65 %) manifested unilateral headache attacks (Table 1).

### Topographic patterns of neural network

**Fig. 2:**
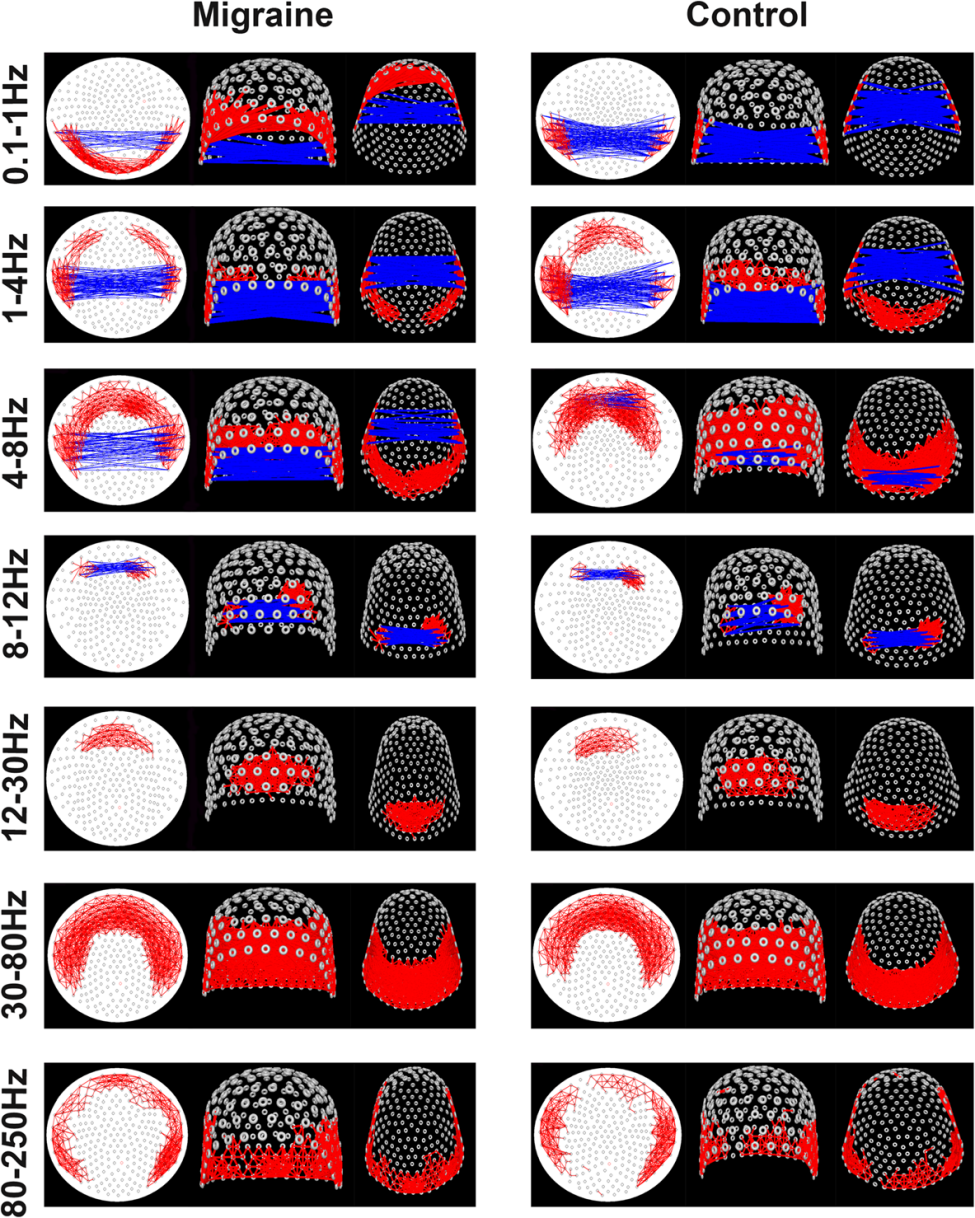
Typical topographic distributions of functional connectivity patterns for seven frequency bands recorded from a migraineur and a control. The red color indicates excitatory connection and the blue color indicates inhibitory connections on contour maps. Compared with the controls, the migraineurs show significantly altered functional connectivity patterns in 0.1–1 Hz. Compared with the MwoA, the MwA shows significantly altered functional connectivity patterns in 4–8 Hz. The MwoA shows the same functional connectivity patterns as the controls in 4–8 Hz

**Fig. 3:**
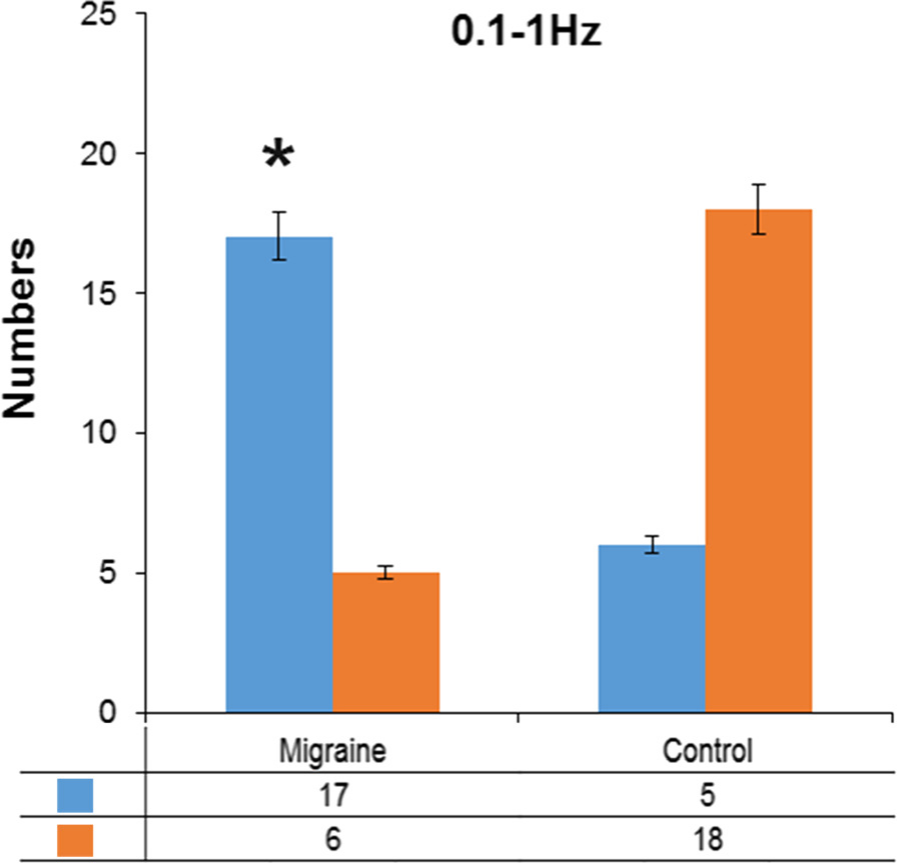
Number of migraineurs and controls in different functional connectivity patterns in 0.1–1 Hz. Migraineurs have significantly higher odds of functional connectivity in the frontal area compared with controls. The blue bars indicate that excitatory connections are present in the frontal area. The orange bars indicate that no excitatory connections exist in the frontal area

In the slow wave (0.1–1 Hz) band, a significant difference was observed in functional connectivity patterns in the frontal cortex between the migraineurs and the controls. Typical topographic distributions of functional connectivity patterns are shown in Fig. 2. The neural network revealed that both the migraine group and the controls had excitatory connections in the left and right temporal sensors, and the connections between the bilateral temporal sensors were inhibitory connections. However, most of the migraineurs (17 of 23) had excitatory connections in the frontal sensors, whereas the controls did not (*p* = 0.001, Fig. 3). This result indicated that the migraineurs had significantly increased functional connectivity in the frontal sensors than that of the controls. No significant difference was observed between the MwA and MwoA in this frequency band.

In the delta (1–4 Hz) band, no significant difference was observed in functional connectivity patterns between the migraineurs and the controls. The neural network showed that both the migraine group and the controls had excitatory connections in bilateral temporal and occipital sensors, and the connections between the bilateral temporal sensors were inhibitory connections. The typical topographic distributions of functional connectivity patterns are shown in Fig. 2. No significant difference was observed between the MwA and MwoA in this frequency band.

**Fig. 4:**
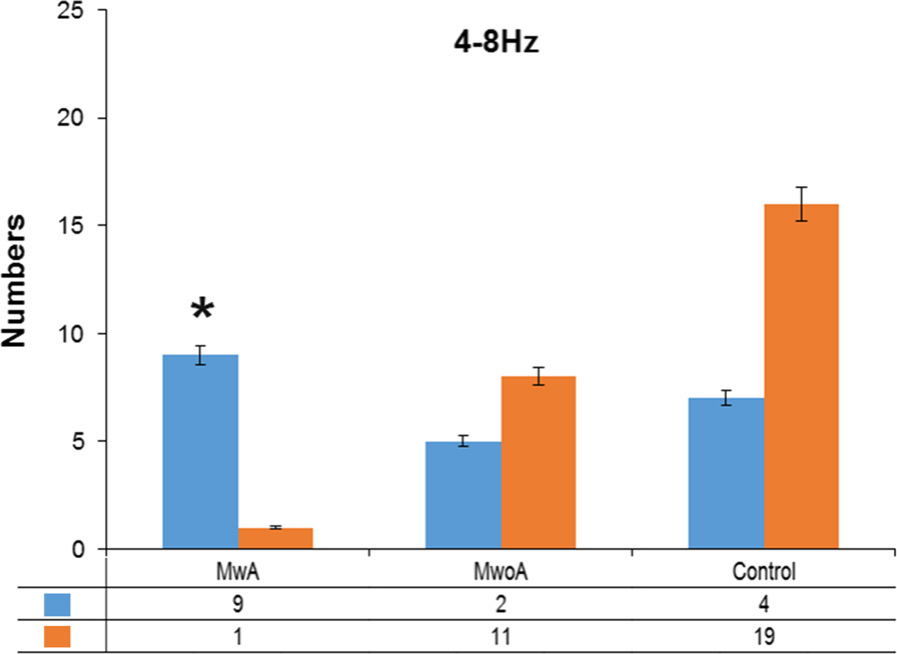
Number of the MwA, MwoA, and controls in different functional connectivity patterns in 4–8 Hz. The MwoA and controls have significantly higher odds of inhibitory connections in the occipital area compared with the MwA. The blue bars indicate that no inhibitory connections exist in the occipital area, and the orange bars indicate that inhibitory connections exist in the occipital area

In the theta (4–8 Hz) band, no significant difference was observed between the MwoA and the controls. The neural network revealed that both the MwoA and the controls had excitatory connections in bilateral temporal and occipital sensors as well as inhibitory connections between the bilateral occipital sensors. However, differences were found between MwA and MwoA. The neural network revealed that the MwA had excitatory connections in bilateral temporal and occipital sensors. However, most MwA (9 of 10) had no inhibitory connections between bilateral occipital sensors and had inhibitory connections between bilateral temporal sensors (*p* = 0.007, Fig. 4). This result may indicate that the MwA had significantly increased functional connectivity between the bilateral occipital sensors than that of the MwoA and the controls. The typical topographic distributions of functional connectivity patterns are shown in Fig. 2.

In the alpha (8–12 Hz) band, no significant difference was observed in functional connectivity patterns between the migraineurs and the controls. The neural network revealed that both the migraineurs and the controls had excitatory connections in bilateral occipital sensors, and the connections between the bilateral occipital sensors were inhibitory connections. The typical topographic distributions of functional connectivity patterns are shown in Fig. 2. No significant difference was observed between the MwA and MwoA in this frequency band.

In the beta (12–30 Hz) band, no significant difference was observed in functional connectivity patterns between the migraineurs and the controls. The neural network revealed that both the migraineurs and the controls had excitatory connections in bilateral occipital sensors. The typical topographic distributions of functional connectivity patterns are shown in Fig. 2. No significant difference was observed between the MwA and MwoA in this frequency band.

In the gamma (30–80 Hz) band, no significant difference was observed in functional connectivity patterns between the migraineurs and the controls. The neural network revealed that both the migraineurs and the controls had excitatory connections in bilateral temporal and occipital sensors. The typical topographic distributions of functional connectivity patterns are shown in Fig. 2. No significant difference was observed between the MwA and MwoA in this frequency band.

In the ripple (80–250 Hz) band, no significant difference was observed in functional connectivity patterns between the migraineurs and the controls. The neural network revealed that both the migraineurs and the controls had excitatory connections in bilateral temporal and occipital sensors. The typical topographic distributions of functional connectivity patterns are shown in Fig. 2. No significant difference was observed between the MwA and MwoA in this frequency band.

### Graph theory analysis

#### Strength

**Fig. 5:**
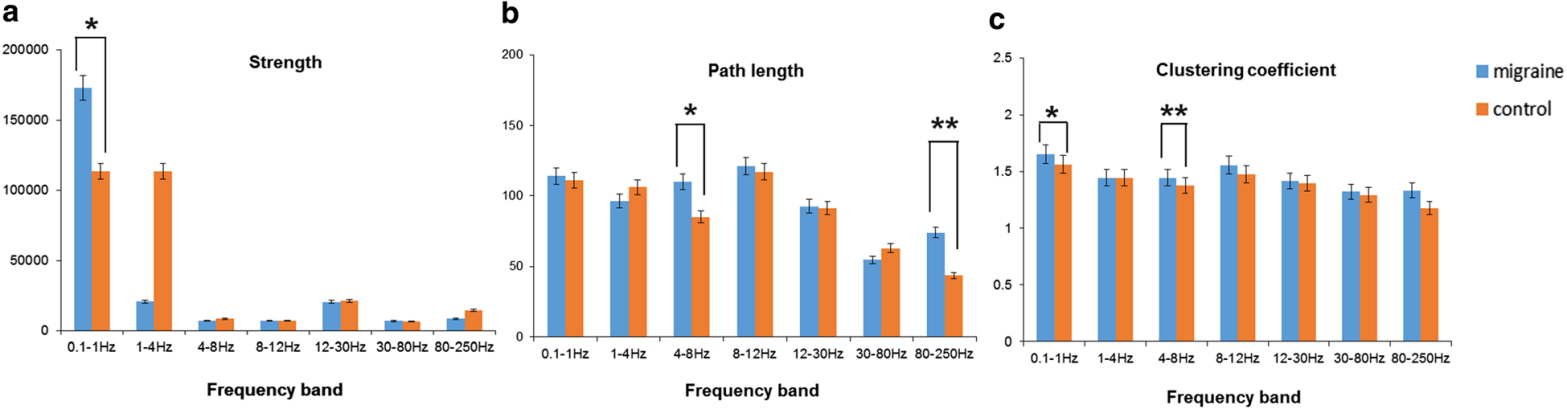
Diffrences in network parameters (strength, path length, clustering coefficient) between the migraineurs and controls. **a** Comparison of the strength of each frequency band of the migraineurs and controls. **b** Comparison of the path length of each frequency band of the migraineurs and controls. **c** Comparison of the clustering coefficient of each frequency band of the migraineurs and controls (*p < 0.016, **p < 0.005)

Group comparison revealed that the average connection strength of the functional connectivity network of the slow wave (0.1–1 Hz) band in migraineurs significantly increased compared with that of the controls (*p* = 0.008). No significant difference was observed between the migraineurs and controls in the other frequency bands. The average connection strength for migraineurs and controls in seven frequency bands are shown in Fig. 5.

#### Path

Group comparison revealed that the average path length of functional connectivity network in migraineurs significantly increased as compared with controls in the theta (4–8 Hz) (*p* = 0.013) and ripple (80–250 Hz) (*p* = 0.002) bands. No significant difference was observed between the migraineurs and controls in the other frequency bands. The average path length for migraineurs and controls in seven frequency bands are shown in Fig. 5.

#### Clustering coefficient

Group comparison revealed that the average clustering coefficient of functional connectivity network in migraineurs significantly increased compared with controls in the slow wave (0.1–1 Hz) (*p* = 0.005) and theta (4–8 Hz) bands (*p* = 0.003). No significant difference was observed between migraineurs and the controls in the other frequency bands. The average clustering coefficient for migraineurs and controls in seven frequency bands are shown in Fig. 5.

### Clinical correlates

Results of correlation analysis demonstrated that there were no significant correlations between migraine clinical characteristics (age, headache history, attack frequency, duration, pain intensity and time from the last attack) and topographic patterns of neural network (*p* > 0.05). There were no significant correlations between migraine clinical characteristics (age, headache history, attack frequency, duration, pain intensity and time from the last attack) and the network parameters (strength, path length and clustering coefficient) (*p* > 0.05).

## Discussion

This study has investigated the functional connectivity neural networks in migraineurs from low- (0.1–1 Hz) to high- (80–250 Hz) frequency ranges by using MEG. The functional connectivity topographic patterns and the measurements of graph theory analysis revealed neuromagnetic differences between the migraineurs and controls in distinct frequency ranges.

Most previous studies have investigated migraine by using MEG while patients were performing a task, including visual stimuli task [34], auditory stimuli task [28] and motor stimuli task [35]. However, these methods can only explore the stimulus-activated brain regions and not the entire brain. By contrast, the present resting-state study focused on the entire brain.

Previous MEG studies investigating resting-state migraine typically analyze a small frequency range [36]. These studies have resulted in several observations, such as suppression of spontaneous cortical activity, long-duration field changes, and large amplitude waves of several seconds’ duration [37]. This study has focused on the functional brain network in migraineurs in a much wider frequency range, i.e., 0.1–250 Hz, with a high sampling-rate MEG system. By combining multi-frequency analysis of functional connectivity with graph theory method, we were able to systematically reveal the functional organization of brain networks in migraineurs compared with conventional analysis of brain waveforms.

The present data obtained from MEG is in consistent with previous reports with EEG [17] that functional connectivity in migraine is significantly impaired. The topographic patterns of neural network showed that the migraineurs had significantly increased functional connectivity in slow wave band as compared with controls. This finding is supported by previous EEG study [16] which has demonstrated an increase in cortical connections during repetitive painful stimulation. However, previous EEG studies [16, 38] mainly focus on low-frequency brain signals (typically < 30 Hz). Therefore, the study of MEG signals above 30 Hz is novel. We consider the study of functional connectivity of neural network in both low- and high-frequency ranges will be important for migraine study.

The analysis of network topographic patterns shows that the migraineurs had significantly increased functional connectivity in the frontal areas than that of the controls in the slow wave (0.1–1 Hz) band. The frontal cortex is one of the most prominent areas associated with brain abnormalities in migraine patients [39, 40]. A recently published paper using MEG has revealed that migraineurs have a high likelihood of neuromagnetic activity in the lateral frontal cortex compared with controls in many frequency ranges [41]. The frontal lobes are responsible for numerous higher order cognitive functions, including planning, decision making, and abstraction, and thus are a primary candidate for dysfunction in many neurodevelopmental and neuropsychiatric disorders [27]. An interictal increase in resting-state functional connectivity in the frontal area may be related to hyperexcitability in the cerebral cortex in migraine. Previous studies have shown a condition of hyperexcitability of pain pathways within the central nervous system, which is thought to represent a crucial event in the neuropathology of migraine [42]. These findings may facilitate to develop new therapeutic strategies for migraine. For example, the repetitive transcranial magnetic stimulation (rTMS) is thought to be effective and well tolerated in the treatment of migraine with aura [43] because high-frequency rTMS increases and low-frequency rTMS decreases the neural excitability of the stimulated cortex [44–46].

The analysis of network topographic patterns also shows that the MwA had significantly increased functional connectivity in the bilateral occipital areas than that of the MwoA and controls in the theta (4–8 Hz) band. These findings suggest a hyperexcitability of the occipital area in the interictal phase of migraineurs with visual aura. Some previous EEG and fMRI studies have also revealed that the migraineurs with aura exhibited an increased resting-state visual network connectivity [19, 47]. These results support that cortical hyperexcitability may play a key role in the cascade of migraine attacks in MwA. This resting-state MEG finding may represent a functional biomarker that could differentiate patients experiencing the aura phenomenon from MwoA, even between migraine attacks.

Quantitative analysis of network using graph theory has revealed that the migraineurs have disrupted organization of brain networks. The disrupted organization of brain networks can be characterized as (1) increased strength in the slow wave (0.1–1 Hz) band; (2) increased path length in the theta (4–8 Hz) and ripple (80–250 Hz) bands; and (3) increased clustering coefficient in the slow wave (0.1–1 Hz) and theta (4–8 Hz) bands. This finding indicated a less optimised topological organization in the intrinsic whole-brain networks of migraineurs. The strength is the sum of weights of connections in the brain areas [13]. An increase of connection strength may indicate a decrease of variations of inter-regional connectivity network. Increased functional connectivity may support the theory of central hyperexcitability in interictal migraine-related brain. The increased path length and clustering coefficient indicated an increased functional segregation and integration of the network topology in migraineurs at the sensor level. Functional segregation in the brain indicates specialised processing within densely interconnected groups of brain regions, whereas functional integration implies rapid combination of specialised information from distributed brain regions [13]. An increase in path length suggests weaker potential for functional integration among brain regions, whereas a higher clustering coefficient corresponds to increased clustering degree of brain regions. As both changes are characterized by a regular topology, we conclude that brain networks in patients with migraine move toward a regular network organization. This finding can advance our understanding of how repeated headache attacks affect the brain topology in migraineurs.

To identify the clinical significance of aberrant MEG parameters in migraineurs, we further calculated the correlations between the abnormal MEG parameters in migraineurs and migraine clinical characteristics. We did not find significant correlations between the network parameters and clinical characteristics in the present study. We speculate that interictal neuromagnetic signals (MEG data recorded during headache free period) might not be able to reflect the headache abnormalities. We noted a previous fMRI study in migraine [48] showed that the clustering coefficient was positively correlated with the duration of migraine in the patients’ functional networks. The discrepancy between the results of this previous study and the present study can be attributed to the different frequency bands used for analysis. Biases inherent to differences in density among networks or differences in the clinical features of patients with migraine may also affect the results.

We noted that this study has some limitations. The first limitation of this study is the number of participants. Given that high-frequency MEG analysis requires a high sampling rate of MEG data, considerable time is needed to compute these MEG data. This limitation is common to many MEG studies; however, with the development of computer technology, the problem will be solved in the future. Another limitation is that neural network analysis was performed only at the sensor level. Given that many network studies with MEG have been performed only at the sensor level [12, 49, 50], source-level analysis may improve the signal-to-noise of the reconstructed time series. In the future research, source-level analysis will be added to compare the difference between the two methods.

## Conclusions

This study has demonstrated that migraine is associated with frequency-specific alterations of functional connectivity networks. Increased functional connectivity in the frontal area was more than likely to play a role in migraine and the increased functional connectivity of occipital was probably related to visual aura. Furthermore, the migraineurs showed a dysregulated brain topology, characterized by increased strength, path, and clustering coefficient. The findings support the notion that cerebral hyperexcitability plays an important role in the cascade of migraine attacks. This resting-state MEG finding may represent a functional biomarker that could provide valuable insights into the underlying pathophysiology resulting from migraine.
